# Doing Science in Uncertain Times

**DOI:** 10.1371/journal.pbio.0020214

**Published:** 2004-07-13

**Authors:** Mikhail Gelfand

## Abstract

Doing scientific research in post-Soviet Russia is challenging but there are solutions that could prevent the massive brain drain witnessed in recent decades

A scientist living in Russia is often asked two questions: “Why haven't you left?” and “Is it still possible to work there?” The best response to the first question is, “Why should I?”—which either terminates the conversation or leads to a stimulating discussion about the fate of the world. The second question, however, deserves a serious answer. In fact, this is the question that every one of us keeps asking ourselves.

There is no simple answer. The biggest problems we face are brain drain, inadequate infrastructure, and lack of money (or perhaps, lack of money, lack of money, and lack of money). In the Soviet Union, fundamental science was supported to a great extent by military expenditure. Thus, it is not surprising that Soviet physics and mathematics were more successful than other fields, such as biology. In the 1990s, military spending on science declined sharply, although the exact numbers are hard to estimate. This year, the direct funding of science constitutes only 1.78% of Russia's national budget (an additional 0.46% is allocated to the space program), although the law stipulates that this figure should be at least 4%. Still, this funding amounts to 46.2 billion rubles (approximately US$1.6 billion), more than twice the amount spent in 2000. Although this figure looks negligible compared with spending on science in the United States and many European countries, it could still be sufficient to support existing actively working groups at a reasonable level.

## Funding

**Figure 1:**
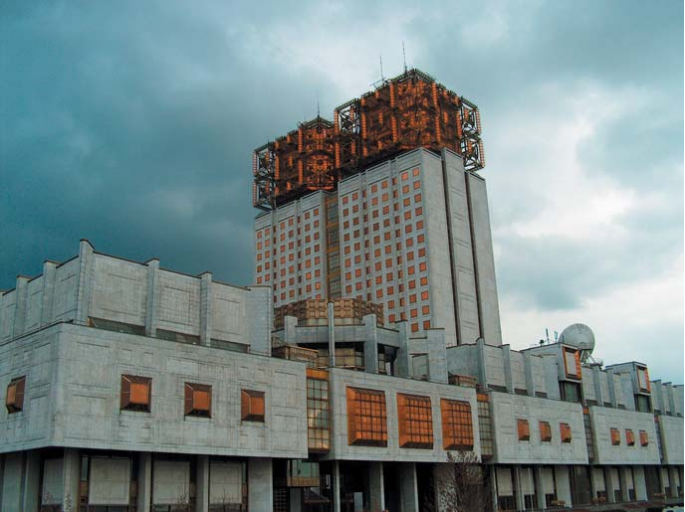
The Golden Brain (the Praesidium of the Russian Academy of Sciences) (Photograph, with permission, by Nataliya Sadovskaya)

There are several mechanisms for distributing funds for research. The major share comes via Russia's Department of Science and the Russian Academy of Sciences. The Academy, unlike its Western analogs, not only acts as a consulting body of experts, but also has the authority to distribute money (Figure 1 [Fig pbio-0020214-g002] [Fig pbio-0020214-g003]). The funds come both as long-term support for scientific institutes and as National or Academy research programs. The former covers base salaries, which are small even by local standards (about US$200 per month for a laboratory chief), and basic infrastructure (water, electricity, etc.). This system of long-term support inherited all the old Soviet ills, such as the lack of correlation between scientific output and the level of funding. As a result, the available resources are spread thinly over hundreds of labs, most of which are just barely alive. The National or Academy research programs can provide funding at a higher level, sometimes even enough to do experimental research. However, the procedure of establishing such programs, though formally competitive, is often not transparent, and a major role is played by the so-called “administrative resource” (Allakhverdov and Pokrovsky 2003).

**Figure 2 pbio-0020214-g002:**
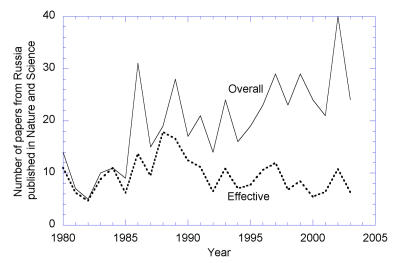
Papers Published in *Nature* and *Science* in Which at Least One of the Coauthors Lists an Address inside the Soviet Union or Russia The actual number of such papers is shown by the solid line. Their “effective number,” into which each paper contributes with the coefficient equal to the fraction of addresses inside the Soviet Union or Russia from all the listed addresses, is shown by the broken line. In recent years, the contribution of ethnic Russians to high-quality research increased, but their work is mostly performed outside Russia.

**Figure 3 pbio-0020214-g003:**
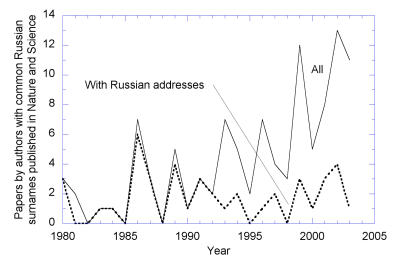
Papers Published in *Nature* and *Science* by Researchers with the 15 Most Common Russian Surnames (Ivanov[a], Kuznetsov[a], Smirnov[a], Etc) The number of all papers in which at least one coauthor has a name from this list is shown by the solid line, and the number of such papers that list at least one address inside the Soviet Union or Russia is shown by the broken line. Whereas before 1992 nearly 100% of ethnic Russians doing toplevel science resided inside the Soviet Union and Russia (hardly surprising!), by now, this number has dropped to below 25%.

Money is also distributed through the Russian Foundation for Basic Research (RFBR). The decisionmaking mechanism used by RFBR is closer to Western standards, and involves anonymous refereeing followed by board discussions. Although its grants are rather small (at most, several thousand dollars per year for a maximum of three years), they provide important additional support for many small and mediumsized groups that may receive several such grants for different projects. In addition, RFBR supports the publication and translation of books, travel to international conferences, the organization of conferences in Russia, and similar activities. Unfortunately, several programs (in particular, support for young scientists) have recently been transferred from RFBR to a newly established government office, and have thus become less independent.

## Collaboration

International collaboration and research grants are a major source of support for many active research groups (Table 1). Several agencies and foundations have programs for Eastern Europe, Russia, and/or former Soviet republics. Some of them, such as the Howard Hughes Medical Institute (Chevy Chase, Maryland, United States), fund individual groups; others—for example the International Science and Technology Center (Moscow, Russia)—stipulate that projects should be submitted jointly by academic and military researchers; and some agencies—in particular, European INTAS (Brussels, Belgium) and the American John E. Fogarty International Center (Bethesda, Maryland, United States)—support collaboration between Russian and Western laboratories.

**Table 1:**
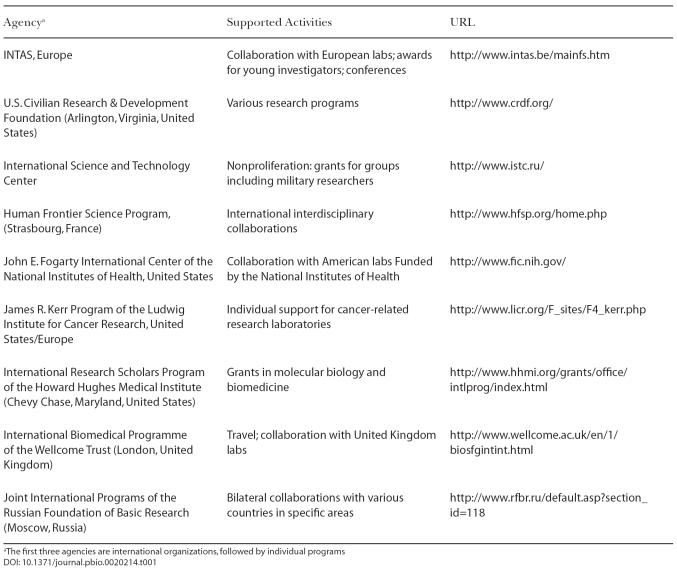
International Agencies Supporting Russian Science

^a^The first three agencies are international organizations, followed by individual programs

Another source of financial support is direct collaboration between Western and Russian laboratories. Even after a relatively short visit, the salary of a visiting researcher abroad can be stretched for several months back home in Russia; even more importantly, experimental biologists visiting foreign labs have access to modern instruments and chemicals, which allows them to do modern research. The hosts of such visits are often (although definitely not always) recent immigrants from Russia, and in such cases the collaborations may have roots in older days (Box 1).

As well as supporting Russian science directly, international collaboration plays an important indirect role because it is less influenced by local politics. In fact, one of the main positive impacts of the New York–based International Science Foundation set up by George Soros in early 1990s was that it demonstrated the possibility of open competition with clearly defined rules—something unheard of in Soviet times—and thus served as a model for the RFBR, which was organized at approximately the same time.

Unfortunately, international ties, especially with the United States, have been adversely affected by recent changes in visa procedures, which have become lengthy (leading to many missed conferences) and, even worse, completely unpredictable (e.g., Brumfiel 2004). The grapevine distributes stories of “bad words” that should be avoided when describing one's research area during an interview at the consulate. Examples of such words include the adjective “nuclear” (even within a innocuous terms like “nuclear magnetic resonance”) or, more recently, anything that involves “bacteria.”

The demand for fundamental and even for applied biological research from Russian industry is almost nonexistent. The pharmaceutical industry is content to produce generics, while Russian biotech companies are still exploiting old strains developed in the Soviet Union. However, some laboratories are conducting outsourced research, and there are now research outposts of Western and Japanese companies in Russia organized as standard industrial labs. On one hand, this work is a dead end for Russian scientists, because the results of such research normally cannot be published. This is a serious problem, especially for young scientists who want to establish themselves. On the other hand, royalties from patents or commercialization of the products can be used to support further research. One group that has followed this path successfully is Sergey Lukyanov's lab at the Shemyakin Institute of Bioorganic Chemistry in Moscow, Russia. They have developed the subtractive hybridization technique for enrichment of clone libraries by rare transcripts or specific genomic fragments (Rebrikov et al. 2004), and are distributing it via a company called Evrogen (http://www.evrogen.com/about.shtml).

## Infrastructure and Bureaucracy

Another major problem is the degradation of infrastructure. Only a few labs can afford modern equipment and instruments, and for many others, even standard chemicals are too expensive. This leads to a vicious circle: without equipment, a lab cannot conduct experiments at the level demanded by highimpact journals—and without such publications, it cannot compete for large grants. Smaller RFBR grants, while simpler to obtain, are insufficient to purchase large pieces of equipment, and funds from several grants or several years cannot be combined due to bureaucratic restrictions. Thus, the only hope for these labs, apart from international collaboration, is a personal connection with senior bureaucrats that might result in an (un)expected windfall.

Having the funds to purchase modern equipment abroad is only the first hurdle; the many conflicting rules and restrictions, inefficiency, and corruption within the system can subsequently hold up the process. Some items, such as tissue samples or animals, are virtually impossible to import legally. The process of clearing the shipments through customs is a difficult, timeconsuming job. Grigory Kopelevich, the Howard Hughes Medical Institute's Russian representative, recalls a story of a grantee whose microscope was stopped at customs because the box contained two screwdrivers not specified in the order. Fortunately, to resolve the issue, it was sufficient to present one of the screwdrivers to a customs officer as a gift.

Even basic access to journals is a problem, especially outside the main research centers. Indeed, out of a random sample of ten major universities where electronic library catalogs were available via the Internet, only six had subscribed to *Nature*, and only two to *Science*. More specialized journals are available only in Moscow and perhaps St. Petersburg. This is partially offset by the proliferating open-access journals from the Public Library of Science and BioMed Central, free electronic versions of older issues provided by some journals, free subscriptions for Russian academic institutes granted by some publishers or purchased by international foundations (e.g., the e-library.ru project organized by the RFBR and supported by the Open Society Institute [the Soros Foundation, based in New York] and the Department of Education) (Table 2), reprints at authors' Web pages, and last but not least, colleagues abroad who break copyright laws by e-mailing PDF files; there is even a popular bulletin board coordinating this activity. However, these are only partial solutions. Russia is not considered a developing country, and thus is excluded from many international efforts that provide free access to journals (such as HINARI). Moreover, many journals have page charges, but no Russian grants cover these, and the cost of publication may be prohibitively high for many groups.

**Table 2:**
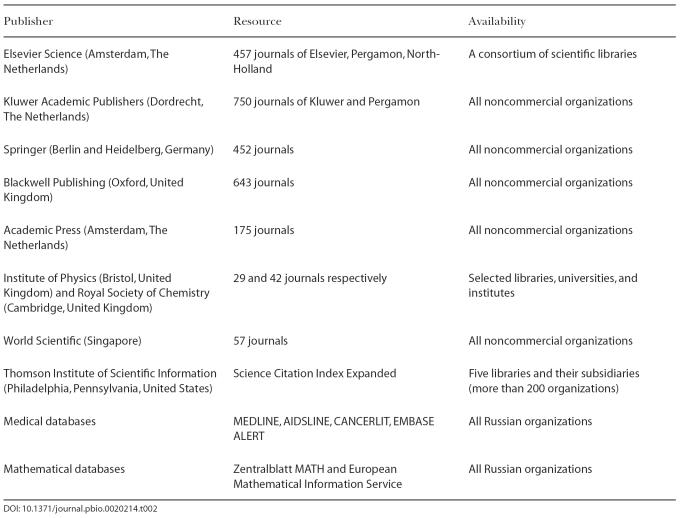
Journals and Other Resources, Available to Russian Academic Institutes under the elibrary.ru Project (e-library.ru)

## Brain Drain

These problems, along with low salaries, have naturally led to a huge brain drain. Entire generations have been decimated (Box 1); the dearth of researchers at the postdoc level, has caused a gap in the teaching and maintenance of scientific traditions. Many labs now consist of older chiefs and senior researchers, and graduate students who plan to leave immediately after getting the candidate degree (the equivalent of a Western doctorate). “Leaving” does not necessarily mean leaving the country; many capable young people go into business. While that might be good for the country in general, it is bad for science, at least in the short term. However, even emigration is not a completely negative thing; it creates a network of collaborators, and in many cases enhances ties with the international community.

Despite all this, science in Russia is very much alive. Not-yet-Nobel-prizewinner Alexei Abrikosov's repeated exhorations to the scientific community “to help all the talented scientists leave Russia and to ignore the rest” were met by universal disgust (Hoffman 1993; Leskov 1993; Migdal 1993). There are several competitive Russian labs doing first-rate research and publishing in the top-tier journals. Old habits die hard; even in these days, very decent results are often published in Russianlanguage journals, the best of which have impact factors that are around 1. Each year, many intelligent and capable students enroll in universities, and competition for admission is steadily increasing from the lows of the mid-1990s. There are also well-attended international conferences in several Russian cities.

## Prospects

What can be done by the international community to support what is left of Russian science? Of course, direct support in the form of competitive grants is important, especially if there are few restrictions on spending; even the most carefully considered procedure cannot foresee all possible situations. But even more useful is the creation of joint research centers, such as the one opened by the international Ludwig Institute for Cancer Research (LICR) based jointly in Zurich, New York, and London, and the Belozersky Institute of Physico-Chemical Biology of Moscow State University in Moscow, Russia. This research center began with limited support for several stronger groups, and is gradually moving toward integration of a research program in Moscow with other LICR projects, and real collaboration between Moscow groups and LICR labs elsewhere.

One of the most essential elements of successful research is access to up-to-date information. Consequently, any initiative that provides open access to scientific literature and databases is extremely useful. Seminars, lecture courses (such as the Moscow University [Moscow, Russia] cycle on oncology and immunology sponsored by LICR; www.oncoimmunology.ru/index_e.htm), and the participation of Western scientists in scientific conferences in Russia are important not only because they provide a fresh understanding of emerging trends, but also because they create personal contacts between Russian and Western scientists that often lead to fruitful collaboration.

By contrast, some other types of joint project may be less successful. Artificial programs aimed at creating various participant “networks” usually do not work as expected, and training programs in Western universities often attract potential emigrants rather that those willing to continue active research inside Russia.

The contribution of the international community cannot be the sole decisive factor in the future growth of Russian science. Important as it is in this transition period, it is no substitute for a systemic change. The ills of Russian science are not unique; the same issues have been raised by scientists from other Eastern European countries (e.g., Wojcik 2004). Even the existing funds could go much further if scientific policies were more open, better structured, and more competitive. Large grants should be provided, on the basis of well-defined criteria, to only the strongest labs doing the best research. An often-heard opinion that no independent review is possible in a small, well-entrenched community is irrelevant, since international boards of experts can be formed—the example of the Soros foundation clearly demonstrates that this is feasible. However, smaller pilot grants are also needed to support young scientists and labs contemplating new projects. This would create competition at all levels and provide doctoral students and postdocs with an incentive to stay in Russia and enroll in a strong lab. But again, the procedure for awarding such grants should be well defined, transparent, and independent from administrative influences.

Thus, the traditional model of top-down distribution of funds must be changed, and this may be difficult. The current system of decision making by Russian funding agencies is clearly inadequate. Moreover, the problems of Russian science mirror the problems of Russian society in general, and it would be naive to expect that they will be solved overnight, even given the political will. Still, if successful, this combination should provide both high-level research in established fields and sufficient flexibility to find new directions.

**Box 1. Top-Level Publications by Russian Scientists**

The vast majority of papers published in recent years in the best journals by scientists working in Russia have foreign coauthors (who are often Russian émigrés), indicating that international collaboration is the most reliable source of support for top-level research. (Text and figures in this box courtesy of Alexey Kondrashov.)

## Abbreviations

LICR: Ludwig Institute for Cancer Research
RFBR: Russian Foundation for Basic Research
